# Effect of intravenous lidocaine on propofol consumption in elderly patients undergoing colonoscopy: a double-blinded, randomized, controlled trial

**DOI:** 10.1186/s12871-022-01601-z

**Published:** 2022-03-04

**Authors:** Meizhen Li, Weiqi Ke, Shaohui Zhuang

**Affiliations:** Department of Anesthesiology, the First Affiliated Hospital of Shantou University Medical College, No. 57 Changping Road, Jinping District, Shantou, Guangdong Province China

**Keywords:** Intravenous lidocaine, Elderly patients, Propofol, Colonoscopy

## Abstract

**Background:**

Elderly patients undergoing colonoscopy with propofol as sedation are prone to respiratory or cardiovascular complications. Intravenous lidocaine has analgesic efficacy and reduces propofol consumption during surgery. Here, the effect of intravenous lidocaine on propofol consumption was evaluated in elderly patients undergoing colonoscopy.

**Methods:**

Patients were randomly allocated to receive intravenous lidocaine (1.5 mg/kg bolus dose, followed by a 2 mg/kg/h continuous infusion during the procedure; Group L) or a placebo (saline; Group N). During the procedure, sedation was achieved by propofol. The following outcomes were recorded: total propofol consumption; time to loss of consciousness; number of airway modifications; time to the first airway intervention; incidence of sedation-related events; pain score after awakening; endoscopists’ and patients’ satisfaction scores; memory level of the procedure; and adverse events within 24 h postoperatively.

**Results:**

Compared with Group N, propofol consumption was reduced by 13.2% in Group L (100.30 ± 25.29 mg vs. 115.58 ± 27.52 mg, respectively, *p* = 0.008). Kaplan–Meier curves showed that the median time to the loss of consciousness episode was shorter in Group L than in Group N (40 s vs. 55 s, respectively, log rank *p* < 0.0001). The number of airway modifications, time to the first airway intervention, incidence of sedation-related events, time to awakening, pain score after awakening, endoscopists’ and patients’ satisfaction scores, memory level of the procedure and adverse events within 24 h postoperatively did not differ between the two groups (*p* > 0.05).

**Conclusions:**

Intravenous lidocaine can reduce propofol consumption in elderly patients undergoing colonoscopy, with quicker time to loss of consciousness.

**Trial registration:**

The clinical trial was registered at (12/01/2021, ChiCTR2100042001).

## Background

Colonoscopy is used for Colorectal cancer (CRC) screening in elderly patients in an increasing number of countries [1]. Propofol has become the first choose sedative drug for colonoscopy [2] because of its rapid action, strong sedation, short half-life, rapid recovery, and higher satisfaction [3]. However, it was reported that respiratory depression and cardiovascular events were common sedation-related complications during propofol-based sedation [4, 5], especially in elderly patients [6]. Moreover, age-related pharmacokinetic changes and patient comorbidities are of great concern during the sedation process. Midazolam and opioid drugs combined with propofol can reduce the consumption of propofol, and both can result in respiratory depression [7, 8]. Therefore, it is of crucial clinical value for anaesthesiologists to find the optimal adjunct drug to reduce propofol consumption and focus on the tolerability of propofol use in elderly patient undergoing colonoscopy.

In recent years, intravenous lidocaine as a propofol-adjuvant drug has been widely used in anaesthesia for reducing pain, decreasing opioid or sedative consumption, and accelerating postoperative bowel function recovery in many surgical procedures [9–11]. Intravenous lidocaine can reduce the requirement of propofol by 50%, with less postoperative pain and fatigue in colonoscopy patients [12]. However, there are no double-blinded, randomized, controlled trials focusing on the use of intravenous lidocaine (1.5 mg/kg, followed by 2 mg/kg/h) in elderly patients undergoing colonoscopy. Therefore, the study aimed to investigate the effect of intravenous lidocaine on propofol consumption in elderly patients undergoing colonoscopy.

## Methods

### Ethical approval of the study protocol

After approval by the Ethics Committee of the First Affiliated Hospital of Shantou University Medical College (No. B-2021-005) and registration on www.chictr.org.cn (12/01/2021, ChiCTR2100042001), this study was performed in the Endoscopic Unit of the First Affiliated Hospital of Shantou University Medical College in Guangdong, China, from January 13, 2021, to May 31, 2021. All the patients who participated in this study voluntarily signed written informed consent forms. This study complied with the Declaration of Helsinki and adhered to the applicable CONSORT guidelines.

### Participants

Patients older than 65 years of age with American Society of Anaesthesiologists (ASA) grading scores of I to III and a body mass index (BMI) from 18 to 30 kg/m^2^ scheduled for colonoscopy were enrolled. The exclusion criteria were as follows: 1) patients with a history of allergies to drugs related to the study; 2) patients with severe heart, lung, liver and kidney diseases; 3) patients with central nervous system diseases or mental disorders; 4) patients with hyperalgesia or refractory cancer pain; and 5) patients who refused to sign the informed consent form.

In addition, during the operation, early procedure termination due to poor intestinal cleansing may have led to less propofol consumption. Patients with an operation time over 40 min were also excluded because patients whose procedure time was more than 40 min were awoken by the endoscopists in the Endoscopic Unit. Endoscopists hope to complete the procedure by changing a patient’s body position. For example, the left lateral decubitus position is commonly changed to the prone position so that the colonoscope may better pass the splenic flexure or the hepatic flexure of the colon. In this case, an anaesthesiologist usually stands by and administrates propofol if necessary. These means that our protocol breaks off at this time, and the total propofol consumption would be different compared to the propofol dose in the study.

### Sample size estimation

Estimation of the sample size was undertaken by PASS 15.0 (NCSS, Kaysville, UT, USA). The primary outcome of this study was the total propofol consumption. Based on the results of our previous study involving 20 patients, the total consumption of propofol for colonoscopy was 124 ± 36 mg in Group N and 106 ± 19 mg in Group L. With a significance level of 0.05 (two-sided) and power of 80%, 42 patients were required in each group. Assuming a loss of follow-up of 8%, 46 patients were required per group, and a total of 92 patients was finally included.

### Randomization and blinding

Ninety-two patients were randomly allocated to the 2 groups. Randomization was determined with block sizes of 4 and an allocation ratio of 1:1. Eligible participants were assigned to receive either intravenous lidocaine (Group L) or normal saline (Group N) according to a computer-generated randomization schedule. This randomization sequence was retained in an opaque envelope. The drugs were prepared by a nurse and administered by an anaesthesiologist who also recorded data. The syringes containing lidocaine or saline were identical because the solutions were clear and colourless. Other individuals cannot identify the solution through its appearance, colour or smell. The patients, endoscopists and anaesthesiologists were blinded to the group allocation.

### Study design

After receiving vascular access in the right upper limb, the patients were placed in the left lateral decubitus position with a nasal cannula oxygen supply of 2 L/minute. The electrocardiogram, peripheral oxygen saturation index (SpO_2_), and noninvasive blood pressure were monitored. All measurements were recorded at intervals of 3 min.

A bolus dose of 1% lidocaine (1.5 mg/kg) was intravenously administered within 2 min before anesthesia induction, followed by a continuous infusion of 2 mg/kg/h (Group L), or the same volume of normal saline was administered (Group N). An initial bolus of propofol (1 mg/kg) was given to all patients. A repeated dose of 0.5 mg/kg of propofol was titrated if necessary to acquire a state of unconsciousness. A dose of sufentanil (0.1 μg/kg) was administered after loss of consciousness. The colonoscope was inserted by an endoscopist after sufentanil was administered. A repeated dose of 0.5 mg/kg of propofol was given if patients expressed discomfort (grimaces, involuntary movements). Propofol was intravenously administered intermittently instead of continuously infused. When subclinical respiratory depression (90% ≤ SpO_2_ < 95%) occurred, the jaw-thrust manoeuvre was performed to open the airway. When hypoxia (75% ≤ SpO_2_ < 90% for less than 60 s) occurred, in addition to the jaw-thrust manoeuvre, the oxygen flow rate was increased from 2 to 6 L/minute. Mask ventilation was performed when severe hypoxia (SpO_2_ < 75% or 75% ≤ SpO_2_ < 90% for 60 s) occurred [13]. Tracheal intubation was necessary if severe hypoxia could not be corrected through mask ventilation, which was decided by the anaesthesiologist. If hypotension (a mean arterial pressure (MAP) < 65 mmHg or a systolic blood pressure (SBP) descending 20% basal value) was continuously measured twice, ephedrine (5 mg) was administered. If bradycardia (heart rate (HR) < 50 bpm) occurred, atropine (0.5 mg) was administered.

The infusion of lidocaine was immediately stopped at the end of the procedure. The procedure time (defined as the time from colonoscope insertion into the anus to withdrawal) was recorded. The satisfaction score for sedation was obtained from the endoscopists. The pain score and memory level were evaluated for patients after awakening. Adverse events within 24 h postoperatively and the patients’ satisfaction scores for the procedure were assessed by telephone for ambulatory patients or interview for inpatients.

### Study outcomes

The primary outcome was the total propofol consumption. The secondary outcomes were as follows: the time to loss of consciousness (defined as the time from intravenous propofol to the loss of the eyelash reflex); the number of airway modifications [5] (defined as opening the airway using the jaw-thrust manoeuvre, increasing the oxygen flow rate or mask ventilation); the time to the first airway intervention; the incidence of sedation-related events [5] (defined as an SpO_2_<90%, hypotension requiring vasopressors, or bradycardia (HR < 50 bpm)); the time to awakening (defined as the time from the final time of intravenous propofol to awakening); pain scores (VAS, visual analogue scale, from 0 to 10; the higher the score, the more intense the pain) after awakening; memory level of procedure (0 = no memory; 1 = memory only at the end; 2 = multiple memories); satisfaction scores (from 0 to 10; the higher the score, the more satisfied) of the endoscopists and patients; and adverse events (such as dizziness, nausea or vomiting) within 24 h postoperatively.

### Statistical analysis

Data were analysed using SPSS 25.0 (SPSS Inc., Chicago, IL, USA) and Prism 9.0 (GraphPad Software, La Jolla, CA, USA). The distribution of variables was evaluated for normality using the Kolmogorov–Smirnov test. Parametric data are presented as the mean ± standard deviation, while nonparametric data are presented as medians with interquartile ranges. Categorical variables are expressed as percentages and were analysed using the χ^2^ or Fisher’s exact test. Continuous variables with a normal distribution were analysed using the two-sample *t* test, whereas continuous variables with a nonnormal distribution were analysed with the Mann–Whitney U test. Kaplan–Meier survival curve analysis with the log-rank test was performed to evaluate the effect of intravenous lidocaine on the time to loss of consciousness. A value of *p* < 0.05 was considered significant.

## Results

**Fig. 1:**
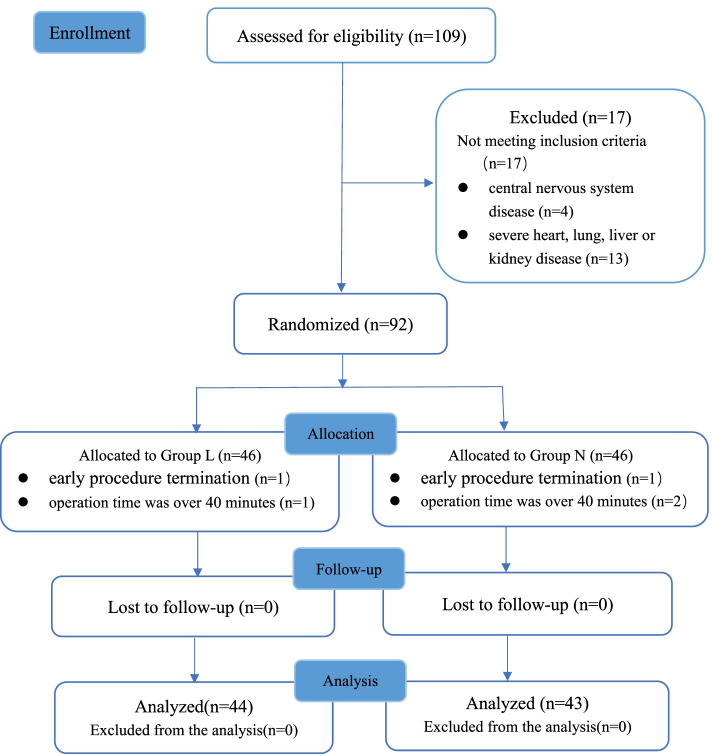
Study population flow diagram

**Fig. 2:**
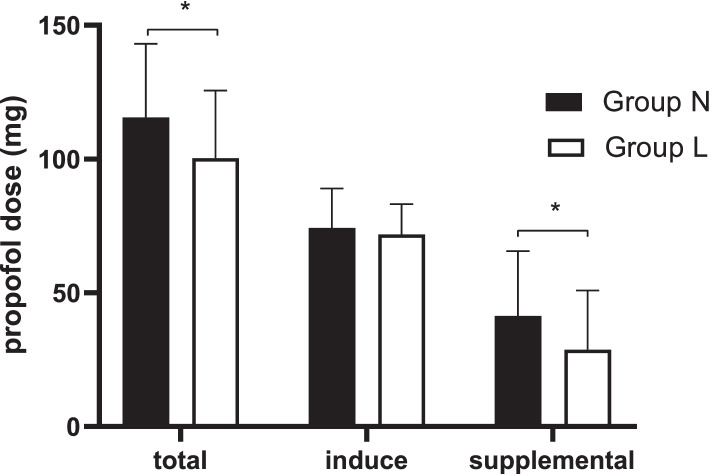
The propofol doses used in the study. Data are the means (SDs). * *p* < 0.05

**Fig. 3:**
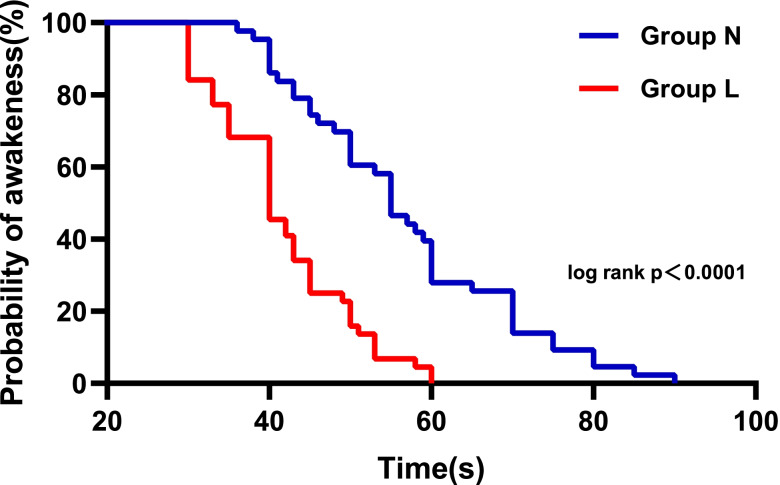
Kaplan–Meier curves for the time to loss of consciousness

Ninety-two patients were included and allocated to either Group L or Group N. One patient in each group were prematurely excluded due to poor intestinal cleansing. One patient in Group L and two patients in Group N underwent a colonoscopy for more than 40 min. Finally, 44 patients in Group L and 43 patients in Group N were enrolled for analysis in this study (Fig. 1). The demographic characteristics and procedure details were not different between the two groups (Table 1). Compared with Group N, propofol consumption was reduced by 13.2% in Group L (100.30 ± 25.29 mg vs. 115.58 ± 27.52 mg, respectively, *p* = 0.008). There was no significant difference in the induction dose of propofol between the two groups, while the supplemental dose was significantly smaller in Group L than in Group N (28.52 ± 22.00 mg vs. 41.35 ± 24.23 mg, respectively, *p* = 0.014) (Fig. 2). The Kaplan–Meier curve showed that the median time to consciousness loss was significantly shorter in Group L than in Group N (40 s vs. 55 s, respectively, hazard ratio = 2.801, 95% CI: 1.742–4.502, *p* < 0.0001) (Fig. 3). The number of airway modifications, time to the first airway modification, incidence of sedation-related events, pain scores after awakening, satisfaction scores of the endoscopists and patients, memory level of procedure and adverse events within 24 h postoperatively did not differ significantly between the two groups (Table 2).

**Table 1:** The demographic characteristics and procedure details

	Group L (*n* = 44)	Group N (*n* = 43)
Age, y	69.00 (66.00–71.75)	69.00 (66.00–73.00)
Sex, Male	28	27
Height, cm	163.43 ± 7.64	162.51 ± 6.76
Weight, kg	59.77 ± 8.69	58.06 ± 8.53
BMI, kg/m^2^	22.38 ± 2.91	21.95 ± 2.64
ASA scores I/II/III	13/30/1	14/24/5
Coexisting disease		
Hypertension	13	8
Diabetes mellitus	13	8
Coronary artery disease	1	1
Main procedure		
Examination	19	14
Polypectomy	18	24
Biopsy	7	5
Procedure time, min	15.11 ± 7.93	16.52 ± 7.12
Ambulatory patient	9	8
1% lidocaine, mg	121.04 ± 23.93	–

*BMI* body mass index, *ASA* American Society of Anesthesiologists

**Table 2:** Secondary outcomes of the study

	Group L (*n* = 44)	Group N (*n* = 43)	*p*
Time to loss of consciousness, s	40[35–48]	55[45–70]	< 0.001
Number of airway modifications, times	1[0–2]	0[0–2]	0.645
Time to the first airway intervention, s	76.50[55.7–163.75]	61.50[30.00–78.75]	0.137
Number of sedation-related events, times	24	23	0.896
Time to awakening, min	7.57 ± 2.39	7.79 ± 2.45	0.673
Pain scores, awake (VAS > 0)	4	8	0.198
Patients’ satisfaction scores	10.0[9.0–10.0]	9.0[10.0–10.0]	0.118
Endoscopists’ satisfaction scores	9.6[9.5–10.0]	9.6[9.5–10.0]	0.698
Memory level			0.749
0, no memory	36	34	
1, memory only at the end	6	8	
2, multiple memories	2	1	
Adverse events within 24 h			
Dizziness	11	4	0.053
Nausea/vomiting	1	0	> 0.999

*VAS* visual analogue scale

## Discussion

This study demonstrated three key findings. First, intravenous lidocaine can reduce propofol consumption in elderly patients undergoing colonoscopy. Second, the time for patient loss of consciousness was significantly decreased by intravenous lidocaine administration. Third, there was no evidence of significant lidocaine–related side effects in elderly patients with this dose of intravenous lidocaine administration.

In this study, intravenous lidocaine resulted in a 13.2% reduction in propofol requirements for colonoscopy. The propofol-sparing effect of lidocaine was not observed during the induction of the anesthesia process, whereas this sparing effect was only observed during surgical stimulation, which suggested that lidocaine mediated an antinociceptive action [14, 15]. The supplemental dose of propofol was significantly lower after intravenous lidocaine administration, which was consistent with the findings of Forster and his colleagues [12]. However, Forster et al. found that using intravenous lidocaine in colonoscopy reduced propofol consumption by 50%, which was significantly higher than the reduction of propofol in this study. Moreover, the procedure time in Forster’s study was obviously longer (25.7 vs. 12.2 min). It was reported that the dose of the sedative was impacted by a variety of factors, including the procedure type and time and the patient’s age, ASA status, and comorbidities [16]. In this study, these factors did not differ significantly between the two groups. Thus, the procedure time in this trial might be the main factor impacting the dose of the sedative used in this trial. The procedure time in this study might have been too short to observe the propofol-sparing effect of intravenous lidocaine. This may partly explain why a 15 mg reduction in propofol might not be meaningful in clinical situations, although it was statistically significant. Meanwhile, the satisfaction scores of the patients and endoscopists did not differ significantly between the two groups, which suggested that the propofol-sparing effect of intravenous lidocaine was not at the expense of the endoscopists’ working conditions.

Abdominal pain, a common complication of colonoscopy, is associated with swelling of the intestinal cavity caused by water and gas injections or mechanical stretching of the intestinal wall caused by the enteroscope during colonoscopy [17]. The analgesic mechanism of intravenous lidocaine is complex and still remains unclear. This finding might correlate with the well-known voltage-gated open and inactivated sodium channel blockade effect [18, 19]. In addition, lidocaine can directly stimulate opiate receptors while suppressing polysynaptic reflexes in the spinal dorsal horn [20, 21]. The pain scores in this study did not differ after intravenous lidocaine, which was different from those in previous research by Forster [12] and Liu [22]. A possible explanation for this observation might be that the continuously infused dose of lidocaine was larger in the two previous studies. Meanwhile, the use of sufentanil also contributed to an analgesic effect. Additionally, the endoscopists extracted the gas from the bowel when the enteroscope was withdrawn from the ileocecum, which might relieve bowel distension. Therefore, the analgesic effect of intravenous lidocaine may be hidden.

Kaplan–Meier and log-rank test analyses showed that the median duration time of consciousness loss in Group L was decreased by approximately one-third, which was similar to that in the research by Liu [22] and Li [23]. Lidocaine combined with propofol shortened the duration of consciousness loss. However, lidocaine did not affect the effect-site concentrations of propofol required for consciousness loss, and the plasma concentration of propofol was not measured in this study. There seems to be a potential pharmacokinetic interaction between these two drugs, which needs to be clarified in future research.

Our results showed that the number of airway modifications and the incidence of sedation-related events did not differ significantly between the two groups. The potential clinical benefits of reducing the propofol dosage when combined with intravenous lidocaine were not ascertained. However, Li et al. [23] demonstrated that compared with propofol alone, lidocaine combined with propofol could significantly reduce the number of oxygen desaturation and apnea episodes in obese patients during colonoscopy, while the hemodynamic parameters during the procedure were similar between the two groups. Interestingly, another study from Chen et al. [24] clarified that lidocaine combined with propofol increased hemodynamic stability in elderly patients when compared with propofol alone, while the SpO_2_ index did not differ. It might be that we focused on elderly patients rather than obese patients, who have a higher incidence of hypoxemia. Although the study population of Chen et al. was the same as ours, the continuously infused dose of lidocaine was different. Whether elderly patients could benefit from reduction on propofol-induced adverse events by intravenous lidocaine still requires a larger sample size and multicenter trial to find the answer.

Doses of lidocaine that exceed safe limits might cause neurological or cardiovascular toxicity, which could be dangerous in elderly individuals. In our study, the safe dose of lidocaine was recommended in the 2016 Enhanced Recovery After Surgery guidelines for gastrointestinal surgery [25]. In addition, all the patients in Group L were observed closely during the perioperative period and for 24 h postoperatively, and none of them showed lidocaine-related side effects. The safety of the administration of lidocaine in our study was adequately guaranteed.

There are some limitations in this study. First, this study mostly included patients who were relatively healthy (ASA I ~ III) and did not include high-risk patients (ASA IV) who were absolutely more vulnerable to propofol. Second, the evaluation of the sedation level and dose supplementation only depended on subjective observation techniques. With monitoring tools such as the BIS index or Narcotrend, the administration of propofol might have been more accurate. Third, the blood concentrations of lidocaine were not monitored. Fourth, compared with Group N, propofol consumption in Group L was reduced by 13.2% (100.30 ± 25.29 mg vs. 115.58 ± 27.52 mg, respectively). Assuming an alpha error of 0.05 (two-sided) and a power of 0.8, the sample size was calculated to be 48 patients per group. However, the actual power was approximately 75% in our study, with a total of 87 patients. Thus, this study was underpowered compared to a power of 0.8 that was estimated when we designed this study. Last, the time to awakening did not differ between the two groups, for which we defined the awakening time as that from the final time administering intravenous propofol to awakening. Propofol was intravenously administered intermittently, and continuous infusion made it easier to determine a different awakening time.

## Conclusion

Intravenous lidocaine can reduce propofol consumption in elderly patients undergoing colonoscopy, with quicker time to loss of consciousness. The safety of the administration of lidocaine in our study was adequately guaranteed. However, the potential clinical benefits of the reduction in propofol-induced adverse events were not ascertained.

## Data Availability

The datasets generated during and analyzed during the current study are not publicly available due to institutional restrictions but are available from the corresponding author on reasonable request.

## Abbreviations

CRC: Colorectal cancer
ASA: American Society of Anaesthesiologists
BMI: Body mass index
SpO_2_: Peripheral oxygen saturation index
MAP: Mean arterial pressure
SBP: Systolic blood pressure
HR: Heart rate
VAS: Visual analogue scale
